# The Glutathione Peroxidase Gene Family in *Thellungiella salsuginea*: Genome-Wide Identification, Classification, and Gene and Protein Expression Analysis under Stress Conditions

**DOI:** 10.3390/ijms15023319

**Published:** 2014-02-21

**Authors:** Fei Gao, Jing Chen, Tingting Ma, Huayun Li, Ning Wang, Zhanglei Li, Zichen Zhang, Yijun Zhou

**Affiliations:** College of Life and Environmental Sciences, Minzu University of China, Beijing 100081, China; E-Mails: gaofei@muc.edu.cn (F.G.); wendybj121@126.com (J.C.); ggtg00@163.com (T.M.); lihuayun0331@163.com (H.L.); wzpn123@163.com (N.W.); lizhanglei2008@126.com (Z.L.); zzcjianao1@163.com (Z.Z.)

**Keywords:** *Thellungiella salsuginea*, glutathione peroxidase, salt stress, drought stress, gene family

## Abstract

Glutathione peroxidases (GPX) catalyze the reduction of H_2_O_2_ or organic hydroperoxides to water or corresponding alcohols using reduced glutathione, which plays an essential role in ROS (reactive oxygen species) homeostasis and stress signaling. *Thellungiella salsuginea* (*Eutrema salsugineum*), a relative of *Arabidopsis thaliana*, displays an extremely high level of tolerance to salt, drought, cold and oxidative stresses. The enzymatic antioxidant systems may contribute to the stress tolerance of *T. salsuginea*. In the present study, we aimed at understanding the roles of the antioxidant enzymes in *T. salsuginea* by focusing on the GPX family. We identified the eight *GPX* genes in *T. salsuginea*, and the structure of the *N*-terminal domains indicated their putative chloroplastic, mitochondrial and cytoplasmic location. The exon-intron organization of these genes exhibited a conserved pattern among plant *GPX* genes. Multiple environmental stresses and hormone response related *cis*-acting elements were predicted in the promoters of *TsGPX* genes. The gene and protein expression profiles of TsGPXs in response to high level of salinity and osmotic stresses, in leaves and roots of *T. salsuginea* were investigated using real-time RT-PCR and western blotting analysis. Our result showed that different members of the *GPX* gene family were coordinately regulated under specific environmental stress conditions, and supported the important roles of TsGPXs in salt and drought stress response in *T. salsuginea*.

## 1. Introduction

In cells, reactive oxygen species (ROS) such as superoxide (•O_2_^−^), hydrogen peroxide (H_2_O_2_), and hydroxyl radical (•OH) are produced during basic metabolic processes like respiration and photosynthesis. In addition, environmental stresses can also promote ROS production, leading to oxidative stress [1]. Although ROS was demonstrated to play an important role in signal transduction when cells are exposed to unfavorable conditions [2], accumulated ROS may result in uncontrolled oxidation of various cellular components, leading to free radical-mediated destruction of the cell structure [3], such as membrane and protein modifications. Eukaryotic cells have evolved elaborate and complicated antioxidant systems, including enzymatic and non-enzymatic components, to maintain the homeostasis between ROS production and elimination. The enzymatic antioxidant systems include superoxide dismutase (SOD), catalase (CAT), glutathione peroxidases (GPX), and glutathione-*S*-transferase (GST).

As a large family of antioxidant enzymes, glutathione peroxidases (GPXs) reduce H_2_O_2_ and organic hydroperoxides to water and correspondingly alcohols using reduced glutathione (GSH), inhibit the ROS-induced damage to membrane and protein, and play crucial roles in protecting cells from oxidative damage [4]. GPXs have attracted extensive investigation in animals and human, and animal GPXs were divided into five classes: cytosolic GPX, gastro-intestinal GPX, plasma GPX, phospholipid hydroperoxide GPX (PHGPX), and seleno-independent epididymis GPX [5]. GPX1 was demonstrated to be an important indicator for cardiovascular diseases prognosis [6], however, our understanding of the structure and function of plant GPXs is limited.

At present, *GPX* genes from several plant species, such as *Nicotiana sylvestris* [7], *Citrus sinensis* [8], *Avena fatua* [9], *Arabidopsis thaliana* [10], *Brassica campestris* [11], *Spinacia oleracea* [12], *Helianthus annuus* [13], *Pisum sativum* [14], *Lycopersicon esculentum* [15], *Oryza sativa* [16], *Triticum aestivum* [17], and *Panax ginseng* [18], have been isolated and characterized, however, few genome-wide *GPX* family identification and characterization studies have been reported. Three GPX family studies were performed in *A. thaliana* [19], *Lotus japonicas* [20], and *Populus trichocarpa* [21]; these studies revealed that plant GPX family consists of multiple GPXs with distinct subcellular location and functions, and that members exhibit different tissue-specific expression patterns and environmental stress responses, functioning coordinately in ROS scavenging. These works also highlight the importance of GPX study at the genome-wide level.

*Thellungiella salsuginea* (*Eutrema salsugineum*), a Brassicaceae species closely related to *A. thaliana* [22], can survive in environments with high soil salinity, low temperature, or water deficiency [23,24]. Compared to *A. thaliana*, *T. salsuginea* was more tolerant not only to high salinity but also to oxidative stress [25], and the level of lipid hydroperoxides in *T. salsuginea* leaves under salt stress was remarkably lower than that of *A. thaliana* [26], indicating that the enzymatic antioxidant systems in *T. salsuginea* behaves more efficiently. However, the genetic basis underlying this physiological mechanism of *T. salsuginea* is still not clear. To date, there is no report on expression profiling of GPXs in *T. salsuginea* under abiotic stress conditions. In the present study, we identified eight *GPX* genes in *T. salsuginea* and analyzed their gene structure and promoter sequences. The potential subcellular locations of *TsGPX* genes were predicted. We examined the expression of TsGPX transcripts and proteins in *T. salsuginea* leaves and roots that were exposed to short-term salt and osmotic stresses.

## 2. Results and Discussion

### 2.1. Identification and Characterization of *TsGPX* Genes

We isolated and characterized the *GPX* genes of *T. salsuginea*. We searched the *T. salsuginea* ESTs in GenBank using the coding regions (CDs) of *GPX1*-*8* from *A. thaliana*, a close relative of *T. salsuginea*. As a result, 7, 1, 1, 1, 2, 2, and 1 ESTs were found, which represent putative *TsGPX1*, *TsGPX2*, *TsGPX3*, *TsGPX5*, *TsGPX6*, *TsGPX7*, and *TsGPX8*, respectively (Table S1). We then assembled all ESTs representing the same *TsGPX* genes, and the full length CDs of *TsGPX1*, *TsGPX2*, *TsGPX6*, and *TsGPX8* were obtained. Because the ESTs or the assembled sequences for *TsGPX3*, *TsGPX5* and *TsGPX7* did not cover the 3′ end of the corresponding *TsGPX* genes, we isolated the full length CDs of *TsGPX3*, *TsGPX5*, and *TsGPX7* using 3′ RACE (Tables S2 and S3, Figure S1). Since the EST sequence for *TsGPX4* was not available, the full length CDs of *TsGPX4* was isolated by PCR using degenerate primers (Table S3) designed according to the cDNA sequence of *AtGPX4*.

We further examined all putative *TsGPX* genes in a recent released version of *T. salsuginea* genome in Phytozome (http://www.phytozome.net/). Thirteen *TsGPX* transcripts were found by blasting with the conserved amino acid sequence and the CDs of *GPX1-8* as the query sequences (Table 1). The thirteen *TsGPX* transcripts were transcribed from nine genomic loci, indicating that several transcripts were products of alternative splicing. For example, Thhalv10000311m and Thhalv10000351m were transcribed from *GPX1* locus; Thhalv10001645m and Thhalv10001644m were transcribed from the *GPX3* locus; Thhalv10006271m, Thhalv10006247m, and Thhalv10006248m were transcribed from the *GPX5* locus.

Thhalv10029228m; a *GPX6*-like transcript; encoded a polypeptide identical to the 3′ part of *TsGPX6* (Thhalv10028932m). Thhalv10029228m may be a new *GPX* gene in *T. salsuginea*, since there is no corresponding gene in *A. thaliana* and other plant species. It may only represent a falsely assembled genomic sequence, since no such EST was found in GenBank or the high-throughput transcriptome database established in our lab (unpublished data). Therefore, we excluded Thhalv10029228m from further analysis in the present study, and will address the existence and the function of this gene in future.

**Figure 1.**
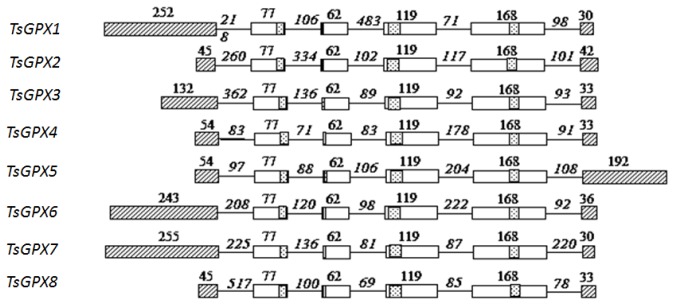
Intron-exon organization of the eight *TsGPX* genes. The box represents exon, and the line represents intron. Dotted boxes show conserved domains, and slashed boxes represent exon 1 and exon 6. The numbers represent the length of the corresponding exon or intron.

All the open reading frames (ORFs) of the eight *TsGPX* genes consisted of six exons and five introns (Figure 1). The same exon-intron organization was also observed for the eight *GPX* genes of *A. thaliana* (*AtGPX*) [19], the six *GPX* genes of poplar [21], and the five *GPX* genes of *L. japonicus* (*LjGPX*) [20], as well as some *GPX* genes of citrus [27] and wheat [17], suggesting a high degree of conservation among plant species in the exon-intron organization.

We also observed a high degree of conservation among plant species in the exon lengths. Exons 2 to 5 have identical size for all the *AtGPX* and *LjGPX* genes, with the exception of exon 5 in *LjGPX4*. The lengths of exon 1 in *TsGPX*, *AtGPX*, and *LjGPX* are variable, since several *GPX* genes lack *N*-terminal signal peptides. The lengths of exon 6 in *TsGPXs* range from 30 to 42 bp, with the exception of *TsGPX5*, which is 192 bp in length (Figure 1). Although most *TsGPX* genes appear to be similar in exon length, the lengths of introns are highly variable; such features are also reported in *GPX* genes from other plant species, such as *Arabidopsis* and *L. japonicas* [19,20].

We noticed that, similar with the other plant *GPX*s, the three conserved domains, “NVASKCG”, “ILAFPCNQF”, and “KWNF (E/T/A) KFLV” [28], were located in the second, third, fourth, and fifth exon, respectively, in all the eight *TsGPX* genes.

Considering that plant *GPX* genes were reported to be involved in responses to various environmental stresses [10,17–19], we analyzed the promoter regions of the eight *TsGPX* genes to locate putative *cis*-acting regulatory elements related to stress and hormone signaling (Table 2) using PlantCARE software [29]. There were multiple *cis*-acting regulatory elements in each *TsGPX* promoters, with different ones in each promoter. The quantity of the *cis*-acting regulatory elements varied with the individual promoter. Almost all *TsGPX* promoters contained *cis*-acting elements that were responsive to methyl jasmonate (*TsGPX2-8*), five *TsGPX* promoters contained elements that are responsive to gibberellin (*TsGPX1*, *2*, *4*, *5*, and *7*), auxin (*TsGPX1*, *3*, *4*, *5*, and *8*) and drought (*TsGPX1*, *2*, *3*, *4*, and *7*), whereas fewer *TsGPX* promoters contained elements involved in response to salicylic acid (*TsGPX1*, *4*, and *7*), low-temperature (*TsGPX1*, *2*, and *5*), and ethylene (*TsGPX6*). We also analyzed the putative *cis*-acting regulatory elements in each *AtGPX* promoter (Table 2). Compared with the *TsGPX* promoters, relatively less stress and hormone response related *cis*-acting regulatory elements were located in *AtGPX* promoters.

### 2.2. Predicted Properties of TsGPX Proteins

**Figure 2.**
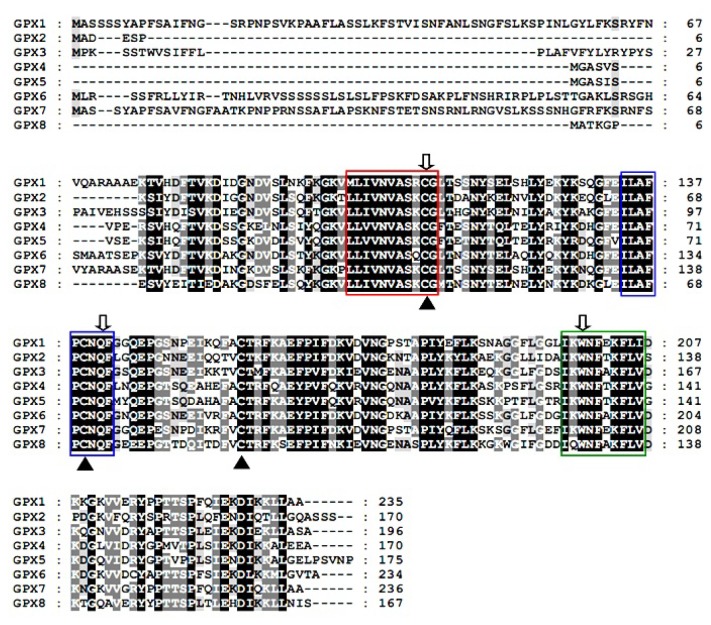
Amino acid sequences of *Thellungiella salsuginea* glutathione peroxidase (TsGPX) proteins. The conserved three Cys residues of plant GPX proteins are indicated by triangles. Boxed sequences represent highly conserved domains in which residues forming a catalytic triad in GPX are marked with an arrow.

The derived amino acid sequences of the eight TsGPX proteins contain not only the three motifs present in most plant GPXs [28], but also other conserved sequences such as NY (T/K/S) (E/Q) L, CT (R/K/M) F (K/Q) (A/S) EFPIF, and PLY (K/E/Q) FLK (Figure 2). These motifs contain the three residues, C (Cys), Q (Gln) and W (Trp), which are proposed to be part of the catalytic site, and the three conserved Cys residues.

**Figure 3.**
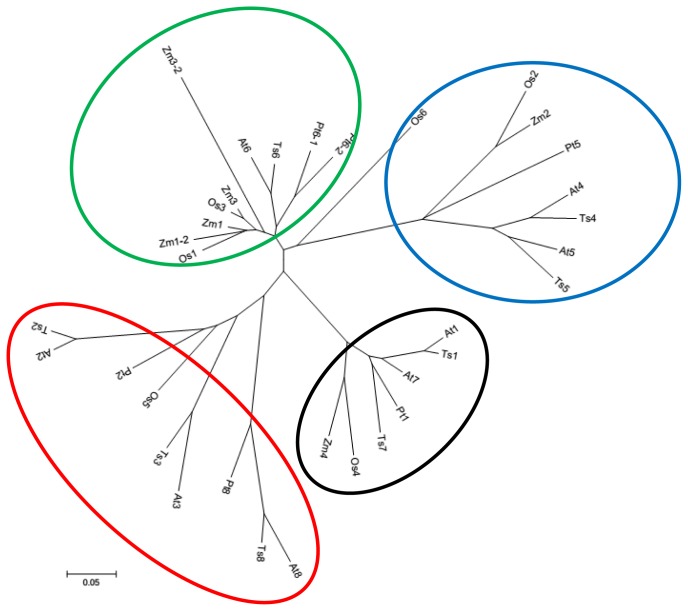
Phylogenetic analyses of thirty four plant glutathione peroxidase (GPX) proteins. The tree was constructed using the neighbor-joining method of CLUSTALW, with 1000 bootstraps, and the bar indicates 0.05 substitutions per site. Each ellipse shows a clade. Abbreviations of plant species: Ts, *Thellungiella salsuginea*; At, *Arabidopsis thaliana*; Os, *Oryza sativa*; Zm, *Zea mays*, Pt, *Populus trichocarpa*. The plant GPXs include At1 (At2g25080), At2 (At2g31570), At3 (At2g43350), At4 (At2g48150), At5 (At3g63080), At6 (At4g11600), At7 (At4g31870), At8 (At1g63460), Pt1 (POPTR_0006s28120), Pt2 (POPTR_0007s02160), Pt5 (POPTR_0014s13490), Pt6-1 (POPTR_0001s09270), Pt6-2 (POPTR_0003s12620), Pt8 (POPTR_0001s09280), Os1 (Os04g0556300), Os2 (Os03g0358100), Os3 (Os02g0664000), Os4 (Os06g0185900), Os5 (Os11g18170), Os6 (A3AYS5_ORYSJ), Zm1 (Q6JAH6_MAIZE), Zm1-2 (B6SU31_MAIZE), Zm2 (B6T5N2_MAIZE), Zm3 (B4FRF0_MAIZE), Zm3-2 (AC204541), and Zm4 (B6U7S4_MAIZE).

According to the alignment of TsGPX sequences shown in Figure 3 and the data presented in Table 3, the eight TsGPX proteins can be divided into three categories based on the amino acid sequence length: TsGPX1, TsGPX6 and TsGPX7 (c. 235 amino acids; 26 kDa); TsGPX2, TsGPX4, TsGPX5, and TsGPX8 (c. 170 amino acids; 19 kDa); and TsGPX3 (196 amino acids; 23 kDa). The poor homology among the *N*-terminal amino acid residues of TsGPX1, TsGPX6, and TsGPX7 indicated that these proteins bear signal peptides for organelle targeting (Figure 3). Protein subcellular localization prediction programs suggested that TsGPX1 and TsGPX7 has a chloroplastic *N*-terminal transit peptide and that TsGPX6 has an *N*-terminal transit peptide for targeting to mitochondria. TsGPX3 was predicted to be located in the secretory pathway (Table 3).

The amino acid sequences of the eight TsGPXs, along with eight AtGPXs, six ZmGPXs, six OsGPXs, and six PtGPXs, were used to build an unrooted phylogenetic tree (Figure 3). The tree is composed of four clades. The black clade included TsGPX1, TsGPX7, AtGPX1, AtGPX7, and PtGPX1. The red clade included TsGPX2, TsGPX3, and TsGPX8, as well as the corresponding *Arabidopsis* GPXs, and PtGPX2 and PtGPX8. The blue clade included TsGPX4, TsGPX5, AtGPX4, AtGPX5, and PtGPX5. The green clade included TsGPX6, AtGPX6, PtGPX6-1, and PtGPX6-2. From this amino acid sequence comparison, it is clear that *Thellungiella* GPXs are more closely related to *Arabidopsis* GPXs than to rice and *Populus* GPXs.

Although TsGPX3 has a relatively larger molecular weight, it was clustered in the same clade as TsGPX2 and TsGPX8, two GPXs with smaller molecular weights, which indicate that TsGPX2 and TsGPX8 are more closely related to TsGPX3 than to TsGPX4 and TsGPX5. The three largest TsGPXs clustered into two clades; TsGPX1 and TsGPX7, two GPXs targeted to chloroplast, were clustered in the black clade, while the mitochondria targeted TsGPX6 was clustered in the green clade. These results are consistent with a previous study [30], and support our subcellular localization prediction for TsGPXs.

### 2.3. Expression Analyses of *GPX* Genes in *T. salsuginea* Plants Exposed to High Salinity and Osmotic Treatment

**Figure 4.**
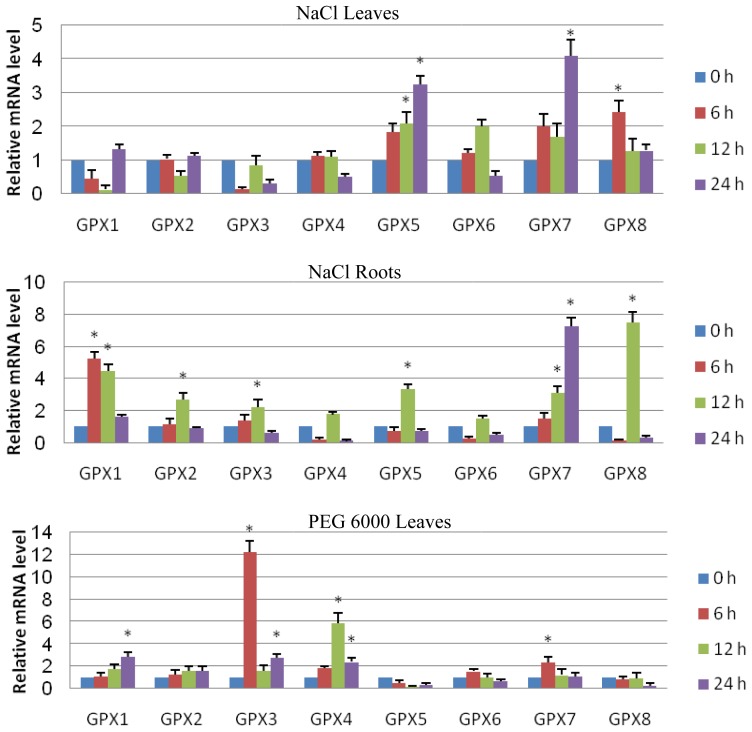

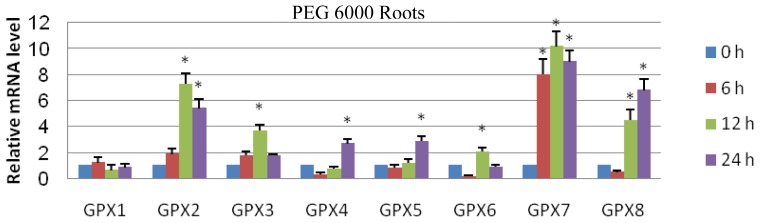
Time-course expression analysis of *Thellungiella salsuginea* glutathione peroxidase (*TsGPX*) genes in leaves or roots of *T. salsuginea* exposed to NaCl (300 mM) or PEG 6000 (20%). mRNA levels were normalized with respect to *TsTubulin* and are expressed relative to the values at 0 h (control), which were given an arbitrary value of 1. Data represent the means ± SE of at least three replicates. Asterisks denote significant up-regulation (ratio > 2.0) or down-regulation (ratio < 0.5).

Considering that multiple stress-response *cis*-acting elements were predicted in the promoters of *Ts GPX* genes, we investigated the expression profiles of *TsGPX* in *T. salsuginea* exposed to high salinity and osmotic stress treatments at the transcriptional level using quantitative RT-PCR technology (Figure 4).

Short-term exposure of *T. salsuginea* plants to salt stress (300 mM NaCl) induced or repressed the expression of some members of *TsGPX* family genes, and the effect varied with tissues, *i.e.*, leaves and roots. In leaves, three *TsGPX* genes (*TsGPX5*, *TsGPX7*, and *TsGPX8*) were highly induced by salt stress at least at one time-point during salt treatment, with the highest transcription level of over 4-fold induced (Figure 4). While in roots, six *TsGPX* genes (*TsGPX1*, *TsGPX2*, *TsGPX3*, *TsGPX5*, *TsGPX7*, and *TsGPX8*) were highly induced by salt stress, with the highest transcription level of over 7-fold induced (Figure 4). Three *TsGPX* genes, *TsGPX5*, *TsGPX7*, and *TsGPX8*, were highly induced by NaCl stress in both leaves and roots, suggesting that they may play important roles in high salinity tolerance in *T. salsuginea* plants.

The expression levels of *TsGPX* family genes were also regulated under short-term osmotic treatment. In leaves, four *TsGPX* genes (*TsGPX1*, *TsGPX3*, *TsGPX4*, and *TsGPX7*) were highly induced by osmotic stress at least at one time-point during treatment, with the highest transcription level of over 12-fold (Figure 4). While in roots, almost all *TsGPX* genes (except *TsGPX1*) were highly induced by osmotic stress, with the highest transcription level of over 10-fold (Figure 4). Three *TsGPX* genes, *TsGPX3*, *TsGPX4*, and *TsGPX7*, were highly induced by osmotic stress in both leaves and roots, suggesting that they may be important in osmotic stress tolerance in *T. salsuginea* plants.

According to the above results, *TsGPX7* may be the most important *GPX* member for salt and osmotic response in *T. salsuginea*, since it was up-regulated by salt and osmotic stress in both leaves and roots. Similarly, the *TsGPX3*, *TsGPX5*, and *TsGPX8* may also play important roles in salt and osmotic response in *T. salsuginea* plants. The functions of these *TsGPX*s in stress tolerance will be investigated in future studies using a transgenic approach.

*T. salsuginea* is an *Arabidopsis*-related extremophile, and analysis of the difference in gene expression between *T. salsuginea* and *A. thaliana* may promote our understanding of how plants cope with abiotic stresses [22,23]. To achieve this goal, we compared the gene expression data of *GPX* genes of *T. salsuginea* to the corresponding data of *A. thaliana* in the AtGenExpress (Table S4) [31]. Under salt stress, *GPX5*, *GPX6*, *GPX7*, and *GPX8* were significantly up-regulated (ratio > 2) in *T. salsuginea* leaves, while *GPX2*, *GPX6*, and *GPX8* were significantly up-regulated (ratio > 1.5) in *A. thaliana* leaves; *GPX1*, *GPX2*, *GPX3*, *GPX5*, *GPX7*, and *GPX8* were significantly up-regulated in *T. salsuginea* roots, whereas *GPX1*, *GPX2*, *GPX4*, *GPX6*, and *GPX7* were significantly up-regulated in *A. thaliana* roots. *GPX5* were found to response to salt stress in *T. salsuginea*, but not in *A. thaliana*, supporting our hypothesis that *TsGPX5* is essential in salt stress response in *T. salsuginea*.

Under osmotic stress, *GPX1*, *GPX3*, *GPX4*, and *GPX7* were significantly up-regulated in *T. salsuginea* leaves, while only *GPX3* and *GPX6* were significantly up-regulated in *A. thaliana* leaves; All *GPX* genes, except *GPX1*, were significantly up-regulated in *T. salsuginea* roots, whereas in *A. thaliana* roots, only *GPX1* was significantly up-regulated, although *GPX6* and *GPX7* were also up-regulated (ratio > 1.4).

The above results reveal that the *TsGPX* family genes exhibit different stress regulated expression patterns from that of *A. thaliana* and more *GPX* genes were induced under salt and osmotic stress conditions in *T. salsuginea*. This is consistent with the *cis*-acting elements shown in Table 2, in which less environmental stress and hormone response related *cis*-acting regulatory elements were found in *AtGPX* promoters. Our results support the hypothesis about the high stress tolerance of *T. salsuginea,* and that differences in salt tolerance mechanisms between salt-sensitive glycophytes, such as *A. thaliana*, and salt-tolerant halophytes, such as *T. salsuginea*, are suggested to result from changes in the regulation of the same basic set of genes involved in salt tolerance [32,33].

However, the *GPX* gene expression difference between *T. salsuginea* and *A. thaliana* should be explained cautiously, since the experiments are carried out in different conditions, and the stress treatment methods were also not identical.

### 2.4. Protein Expression Analyses of *GPX* Genes in T. salsuginea Plants Exposed to High Salinity and Osmotic Treatments

We further conducted Western blotting analysis to investigate the protein expression level of *TsGPX* genes under stress conditions, since the gene expression level is not always correlated with protein abundance [34]. The antisera against unique polypeptide sequence of each TsGPX proteins (Table S5) were prepared through rabbit immunization. It is noteworthy that, because of the high sequence similarity between TsGPX4 and TsGPX5, one antiserum was prepared and used for detecting both proteins. The TsGPX8 cannot be detected in leaves, which may be due to its low abundance in leaves.

**Figure 5.**
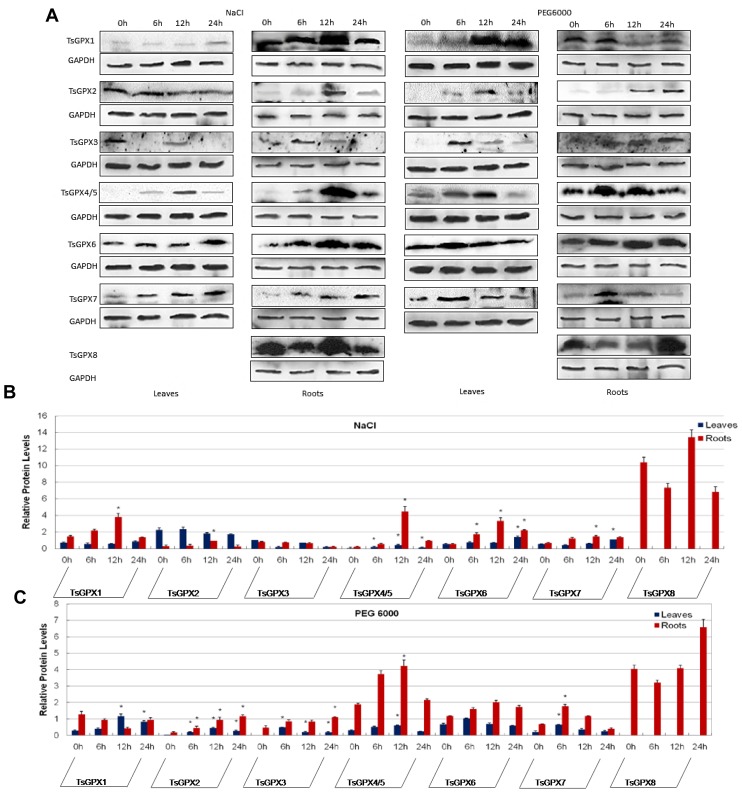
Western blotting and quantification. (**A**) Western blotting analysis of *Thellungiella salsuginea* glutathione peroxidase (TsGPX) in leaves or roots of *T. salsuginea* before (0) and at 6-, 12-, and 24-h after NaCl or PEG 6000 treatment. Anti-OsGAPDH antibody was used as a control for loading error; (**B**) Bar graph for the quantitative comparison among TsGPX protein expression levels during NaCl treatment; (**C**) Bar graph for the quantitative comparison among TsGPX protein expression levels during PEG 6000 treatment. TsGPX- and OsGAPDH-specific bands were quantified by ImageJ software and TsGPX protein expression levels were normalized to GAPDH values. Mean values ± SD of 3 independent experiments are shown. Asterisks denote significant up-regulation (ratio > 2.0) or down-regulation (ratio < 0.5).

Short-term salt stress affected the protein abundance of some members of the TsGPX family. In leaves, TsGPX4/TsGPX5, TsGPX6, and TsGPX7 showed an up-regulated pattern at least at one time-point during salt treatment, reaching their peaks at 24 h (TsGPX6 and TsGPX7), or at 12 h (TsGPX4/TsGPX5) (Figure 5). While in roots, almost all TsGPXs (except TsGPX3) were induced at least at one time-point during salt treatment, reaching their peaks at 12 h.

Western blotting analysis also revealed that the protein expressions of TsGPX2, TsGPX3, TsGPX4/TsGPX5, and TsGPX7 were induced under short-term osmotic treatment in both leaves and roots, although their abundance peaks emerged at different time-points. For example, the abundance of TsGPX7 reached peak at 6 h in both leaves and roots, while TsGPX2 reached peak at 12 h in leaves and 24 h in roots.

Quantitative real-time RT-PCR is a widely used method for monitoring gene expression at the transcriptional level, while Western blotting is a widely accepted analytical technique to detect specific proteins in a sample of tissue extract. The regulation patterns of TsGPXs in *T. salsuginea* plants under stress conditions as revealed by both approaches is consistent in general, with some minor differences. For example, the *TsGPX3*, *TsGPX5*, *TsGPX7* and *TsGPX8*, four *GPX*s shown to be important for salt and osmotic response in real time RT-PCR analyses, were found to be up-regulated in protein level in most of the stress conditions. It is also noteworthy that the antiserum used in the present study cannot detect TsGPX8 protein in leaves, while the gene expression of *TsGPX8* was determined readily by the quantitative real-time RT-PCR, suggesting that the protein abundance of TsGPX8 may be very low in leaves of *T. salsuginea*.

## 3. Experimental Section

### 3.1. Plant Materials and Stress Treatments

*Thellungiella salsuginea* (Shandong ecotype) seedlings were grown in pots containing a mixture of vermiculite and perlite (1:1, *v*/*v*) under fluorescent light (120 μmol m^−2^ s^−1^, 16 h light/8 h dark) at 25 °C and 35%–50% relative humidity in a growth chamber. The plantlets were watered in a three-day interval with half strength of Hoagland’s solution.

Five-week-old plants were used as the starting materials for stress treatments. The salt treatment was applied by watering the seedling with 300 mM NaCl in half strength Hoagland solution, and the osmotic treatment was applied by watering the seedling with 20% PEG 6000 solution. The leave and root samples were collected 6, 12, and 24 h after treatment. The seedlings before treatment were used as the control (0 h). The tissue samples were immediately frozen in liquid nitrogen, and then stored at −80 °C for extracting RNA and protein. At least three biological repeats from each treatment were prepared for real-time PCR and western blotting analysis.

To monitor the water status of the stressed plants, the relative water contents (RWC) or the water potentials of the leaves were measured according to a method described previously [35], or with a WP4 dewpoint potentiometer (Decagon Devices, Pullman, WA, USA), respectively (Figures S2 and S3).

### 3.2. Isolation of the Eight *TsGPX* Genes

We searched the *T. salsuginea* ESTs in the GenBank using the CDs of *GPX1*-*8* from *A. thaliana*. The ESTs representing *TsGPX* genes were assembled using software DNASTAR (DNASTAR Inc., Madison, WI, USA). The primers used for isolating full length CDs of *TsGPX* gene were designed using Primer3 (http://primer3.ut.ee/). Total RNA from *T. salsuginea* seedlings was extracted using TRIzol reagent (Invitrogen, Carlsbad, CA, USA). The potential contaminating genomic DNA was treated with RQ1 RNase-free DNase (Promega, Madison, WI, USA). The cDNA was synthesized using BcaBEST RNA PCR Kit (Takara, Shiga, Japan) according to the manufacturer’s protocol. 3′ RACE were performed to isolate full length CDs of some members of *TsGPX* gene family using 3′ Full RACE Core Set Ver.2.0 kit (TaKaRa, Shiga, Japan). All primers are listed in Tables S2 and S3.

### 3.3. Sequence Alignments, Phylogenetic Analysis, and Other Bioinformatics Analysis

Molecular weight (MW) and isoelectric point (pI) predictions for each deduced TsGPXs were performed using the Compute pI/Mw tool (http://www.expasy.org/tools/protparam.html). The deduced sequences of eight TsGPXs were aligned with the DNAMAN program (version 4.0; Lynnon Corporation, Quebec, Canada) to identify conserved domains. These TsGPXs were also used, together with the sequences of the homologue proteins from other green plants, to construct an unrooted phylogenetic tree by the neighbour joining method with the ClustalW1.8 [36] and MEGA 6.0.5 [37] programs. Intron-exon organization of *TsGPX* genes were analyzed using an online tool, Spidey (http://www.ncbi.nlm.nih.gov/spidey/). Predictions of transit peptides and subcellular localization were conducted with the programs TargetP1.1 [38] and PSORT [39].

For promoter analysis, we first used Neural Network Promoter Prediction [40] (http://www.fruitfly.org/seq_tools/promoter.html) to locate the transcription start site in the 2 kb upstream region from translation start code ATG, and then predicted *cis*-acting elements using PlantCARE (http://bioinformatics.psb.ugent.be/webtools/plantcare/html/) [29].

### 3.4. Quantitative Real-Time RT-PCR

Approximately 1 μg of DNase I-digested total RNA was converted into single-stranded cDNA using M-MLV Reverse Transcriptase (Promega, Madison, WI, USA). The cDNA were diluted 50-fold with deionized water before use as a template in real-time PCR. The quantitative reaction was performed on a MyiQ2 two-color real-time PCR detection system (Bio-Rad Laboratories, Hercules, CA, USA) using the SsoFast EvaGreen Supermix (Bio-Rad Laboratories, Hercules, CA, USA). The reaction mixture (20 μL) contained 2× SsoFast EvaGreen Supermix, 0.9 μM each of the forward and reverse primers, and 1 μL of cDNA template. PCR amplification was carried out under the following conditions: 95 °C for 30 s, followed by 40 cycles of 95 °C for 5 s and 60 °C for 10 s. Three independent biological replicates for each sample and three technical replicates of each biological replicate were analyzed in quantitative real-time PCR analysis. The gene expressions of *TsGPXs* were normalized against an internal reference gene, *TsTubulin* (Accession number: BY821407). The relative gene expression was calculated according to the 2^−ΔΔCt^ method [41]. All primers used in this study are listed in Table S6. The specificity of primers was verified using amplicon dissociation curves and gel-electrophoretic analysis. We also sequenced the PCR products of each pair of primer to validate their specificity.

### 3.5. Antibody Preparation and Western Blotting Analysis

The antisera against TsGPXs were prepared by immunizing rabbits with the polypeptides representing individual TsGPXs (Table S5). One antiserum was prepared and used for detecting both TsGPX4 and TsGPX5, due to their high level of sequence similarity. The titers of antisera against TsGPX1, GPX2, GPX6, GPX4/GPX5, GPX7, and GPX8 were no less than 51,200, and the titer of TsGPX3 was 12,800. We further validated their efficacy using the *E. coli* expressed TsGPXs.

The rosette leaf proteins were extracted using a trichloroacetic acid/acetone method as described previously [42]. The protein concentration was measured using Bradford methods (Bioteke, Beijing, China). Bands on the membrane were finally visualized using a Pro-Light HRP Chemiluminescence Kit (TIANGEN, Beijing, China) and captured using a Tanon 5500 chemiluminescence Gel imaging system (Tanon, Shanghai, China). Anti-OsGAPDH antibody (Beijing Protein Innovation, Beijing, China) was used as the internal reference. TsGPX- and OsGAPDH-specific bands were quantified by ImageJ software (http://rsbweb.nih.gov/ij/).

## 4. Conclusions

In conclusion, we performed a genome-wide identification of the *GPX* family genes in *T. salsuginea*. The gene and protein expression profiles of *TsGPX*s in response to high salinity and osmotic stress, in leaves and roots of *T. salsuginea* were investigated using quantitative real-time RT-PCR and Western blotting analysis, and the difference in stress-induced expression patterns of *GPX* family genes were analyzed. Our results showed that in different tissues, different members of the *GPX* gene family were coordinately regulated under specific environmental stress conditions. The results presented in this study therefore provide evidence at both the transcriptional and protein levels that support the conclusion that GPXs, such as, GPX7, play important roles in salt and osmotic stress response in *T. salsuginea*.

## Supplementary Information



## Figures and Tables

**Table 1.** *TsGPX* genes and their genomic locations.

Number	Sequence ID	Name	Location in genome
1	Thhalv10000311m	*GPX1*	scaffold_15: 4940063–4941983
2	Thhalv10000351m	*GPX1* isoform	scaffold_15: 4940063–4941470
3	Thhalv10017319m	*GPX2*	scaffold_10: 6460958–6462801
4	Thhalv10001645m	*GPX3*	scaffold_22: 63411–66356
5	Thhalv10001644m	*GPX3* isoform	scaffold_22: 63411–66356
6	Thhalv10001660m	*GPX4*	scaffold_22: 1860223–1861484
7	Thhalv10006271m	*GPX5*	scaffold_19: 155838–157677
8	Thhalv10006247m	*GPX5* isoform	scaffold_19: 155838–157955
9	Thhalv10006248m	*GPX5* isoform	scaffold_19: 155838–157677
10	Thhalv10028932m	*GPX6*	scaffold_3: 3990214–3992118
11	Thhalv10029228m	Not determined	scaffold_3: 3955295–3956113
12	Thhalv10026852m	*GPX7*	scaffold_1: 3456273–3457732
13	Thhalv10023725m	*GPX8*	scaffold_8: 965149–966667

**Table 2.** Putative *cis*-acting regulatory elements related to stress and hormone response in *TsGPX* and *AtGPX* promoters.

Environmental stress or hormone	*Cis*-acting regulatory elements	Sequence	*TsGPX*	*AtGPX*
Abscisic acid (ABA)	ABRE	TACGTG	*TsGPX2*, *4*, *5*, and *7*	*AtGPX1*, 4,*5*, and *8*

Auxin	TGA-element	AACGAC	*TsGPX1* and *8*	*AtGPX2* and *8*
	AuxRR-core	GGTCCAT	*TsGPX3*, *4*, and *5*	*-*

Salicylic acid (SA)	TCA-element	CCATCTTTTT	*TsGPX1* and *4*	*AtGPX4*
		GAGAAGAATA	*TsGPX7*	*AtGPX3* and *7*

Gibberellin (GA)	TATC-box	TATCCCA	*TsGPX1*	*-*
	GARE-motif	TCTGTTG	*TsGPX2*	*-*
	P-box	GCCTTTTGAGT	*TsGPX4* and *5*	*AtGPX5*, and *7*
	GARE-motif	TCTGTTG	*TsGPX5*	*-*
		AAACAGA	*TsGPX7*	*AtGPX3*, *6*, and *7*

Methyl Jasmonate (MeJA)	CGTCA-motif	CGTCA	*TsGPX2*, *3*, *4*, *5*, *6*, *7*, and *8*	*AtGPX3*, *4*, *5*, *7*, and *8*
	TGACG-motif	TGACG	*TsGPX2*, *3*, *4*, *5*, *6*, *7*, and *8*	*AtGPX3*, *4*, *5*, *7*, and *8*

Ethylene	ERE	ATTTCAAA	*TsGPX6*	*AtGPX1*, *3*

Drought inducibility	MBS	TAACTG	*TsGPX1*, *2*, *3*, *4*, and *7*	*AtGPX2*, *5*, *7*

Low-temperature	LTR	CCGAAA	*TsGPX1*, *2*, and *5*	*AtGPX5* and *8*

Anaerobic induction	ARE	TGGTTT	*TsGPX2*, *3*, *6*,and *8*	*AtGPX2*, *3*, *7*, and *8*

**Table 3.** The physical and chemical properties of TsGPXs.

Name	Length (aa)	Molecular mass (kDa)	pI	TP *	Subcellular localization *	Transmembrane domain *
TsGPX1	235	25.902	9.94	73	chloro	no
TsGPX2	170	19.020	5.48	–	cyt	no
TsGPX3	196	23.258	7.33	–	SP, CM, ER	yes
TsGPX4	170	19.147	9.08	–	cyt	no
TsGPX5	175	19.612	9.79	–	cyt	no
TsGPX6	234	25.937	9.35	58	mito	no
TsGPX7	236	26.227	10.30	75	chloro	no
TsGPX8	167	18.990	4.75	–	cyt	no

* Transit peptides (number of amino acid residues) and subcellular localizations (mito, mitochondria; chloro, chloroplasts; cyt, cytosol; SP, secretary pathway; ER, endoplasmic reticulum; CM, cytoplasmic membrane) of TsGPX proteins, as predicted by the TARGETP and PSORT programs.
