# Supplementary material for: The Glutathione Peroxidase Gene Family in Thellungiella salsuginea: Genome-Wide Identification, Classification, and Gene and Protein Expression Analysis under Stress Conditions
Source: Int J Mol Sci. 2014 Feb 21;15(2):3319–35. doi: 10.3390/ijms15023319 (PMC3958914; doi:10.3390/ijms15023319)
Supplement: Supplementary file 1 [file ijms-15-03319-s001.pdf]

Supplementary Information

Table S1. *TsGPX* genes related ESTs in GenBank.

Gene name	Number of ESTs	Accession number
TsGPX1	7	BY805042, BY834089, BY814638, BY831158, BY805041, BY831159, BY834088
TsGPX2	1	FJ357243
TsGPX3	1	BY825912
TsGPX5	1	BY818373
TsGPX6	2	BM985497, BM986105
TsGPX7	2	BY816539, DN774281
TsGPX8	1	BY806901

Table S2. Gene specific primers used in 3' RACE.

Gene name	Primer sequences (5'–3')
*TsGPX3*_SP1	CCTTATTCTCCAGCCATCGT
*TsGPX3*_SP2	GGAATGATGTGAGTTTGAGCCAG
*TsGPX5*_SP1	CCGAGAAATCCATCCACCAA
*TsGPX5*_SP2	GCTTCTAAATGCGGTTTCACG
*TsGPX7*_SP1	AACCAAACCCAATCCACCTCG
*TsGPX7*_SP2	ATGTCAATGGACCGAGCAC

Table S3. Primers used for isolating the CDs of *TsGPX* genes.

Gene name	Forward primers (5'–3')	Reverse primers (5'–3')
TsGPX1	ATGGCTTCTTCTTCTTCTTACGCAC	CCATGACCGGTAACATTAAGATTC
TsGPX2	CTCAGAAAGAGACCAACGACAC	CATCCATGAAACGGCATCTCAACAATG
TsGPX3	TGTCGATGCCTAAATCAAGC	GAAAATGAGATTCACACTGGTACTC
TsGPX4	GGGAGATAGAGATGGGTGCT	CATGATTAACCGGGATGGTG
TsGPX5	GAGGACAAAACTGGCGAGAG	CAATTCGGAGAGTCGTAGCC
TsGPX6	GCAAATGCTTCGCTCCTCAATTCG	CCCAAATACACTCAAGAACTCAGACAG
TsGPX7	GTAATGGCTTCCTCTTACGCACCA	TACGATGCACACATGGGAAC
TsGPX8	CGTCTCTCATTTCTCCTGCTTCTG	GGAGATATTCAGAAGCTTCTTTATGTC

Table S4. *AtGPX* genes expression under salt and drought stresses. The data were manually extracted from AtGenExpress. Highlighted data denote significant up-regulation (>1.5-fold).

Salt stress: 150 mM NaCl				
**Shoot**				
	0 h	6 h	12 h	24 h
GPX1	10064.4044	6895.3897	2701.4183	6239.0952
GPX2	5246.3739	5447.4280	3024.1973	9346.0837
GPX3	159.9263	219.3388	213.3176	207.4407
GPX4	7.4473	6.6617	6.1240	7.0386
GPX5	323.0878	430.1305	389.1488	416.5335
GPX6	541.5357	3902.5248	2696.5284	1118.0756
GPX7	1279.2264	118.8702	28.1226	1058.7937
GPX8	290.7771	428.4135	458.2031	346.8564
**Root**				
	0 h	6 h	12 h	24 h
GPX1	389.8374	223.8204	450.2026	590.9868
GPX2	5586.5607	3906.6186	7840.4588	6560.5704
GPX3	294.3101	153.1334	256.1301	269.7614
GPX4	8.1259	14.5233	8.1488	10.6864
GPX5	653.3744	432.7572	509.1552	567.7098
GPX6	3112.7230	5896.1167	5051.1119	3453.4035
GPX7	10.5994	12.9030	14.6064	14.1410
GPX8	1643.8376	643.7455	934.3056	982.2966
**Drought stress**				
**Shoot**				
	0 h	6 h	12 h	24 h
GPX1	10064.4044	8515.1334	3248.3612	12107.0491
GPX2	5246.3739	2759.6138	2582.8341	3981.7766
GPX3	159.9263	298.8367	249.6073	154.7041
GPX4	7.4473	5.2068	7.1161	7.6206
GPX5	323.0878	412.5666	386.3245	330.3208
GPX6	541.5357	2626.6964	1635.7669	368.6772
GPX7	1279.2264	54.3319	25.7462	805.2453
GPX8	290.7771	399.8747	494.3356	200.2977
**Root**				
	0 h	6 h	12 h	24 h
GPX1	389.8374	572.1580	364.2638	457.8970
GPX2	5586.5607	4586.6898	4651.6141	3580.1164
GPX3	294.3101	350.1968	304.0444	287.5815
GPX4	8.1259	6.3090	6.9119	6.6159
GPX5	653.3744	507.4506	487.5507	587.0016
GPX6	3112.7230	4358.5389	3790.3118	2432.0862
GPX7	10.5994	15.3150	10.7799	13.6470
GPX8	1643.8376	1413.9898	1161.6843	1242.5744

Table S5. The synthetic peptides used for TsGPXs antibodies preparation.

GPXs	Peptide sequences
TsGPX1	FNGSRPNPSVKPAAFLASC
TsGPX2	SPDGKVFQRYSPRTSPLC
TsGPX3	FVFYLYRYPYSPAIVEHC
TsGPX4/TsGPX5	CLVGKDGQVIDRYGPT
TsGPX6	CTTGAKLSRSGHSMAATS
TsGPX7	CPSKNFSTETSNSRNLRNGV
TsGPX8	DKTGQAVERYYPTTSPLTLC

Table S6. Primers used for quantitative real-time RT-PCR analysis.

Gene	Forward Primers (5'–3')	Reverse Primers (5'–3')
TsGPX1	ATCAATTTGGTGGCCAAGAG	AAGAATCCTCCTGCGTTTGA
TsGPX2	GGGACAAGAACCAGGAAACA	AGACTTTGCCGTCAGGAGAA
TsGPX3	ACCTGGTAGCAACGAGGAGA	TGGAGCATATCGATCCACAA
TsGPX4	CATTCCCTTGCAACCAGTTT	GGCCGTAACGATCAATCACT
TsGPX5	GAGGACAAAACTGGCGAGAG	AAACGCCAAGATCACAAACC
TsGPX6	TCGTCCTCTTCCTTTATCGACAACG	CGCATCCTTCACGGTGAAATCATAG
TsGPX7	GACCGAGCACAGCTCCTATC	TCTCGATTTGGAAGGGAGAA
TsGPX8	GGCCCAGAATCTGTTTACGA	TGGTCAGTAGTTCCCGGTTC
TsTublin	GTCAGTCTGGTGCGGGTAAC	CCAGATCCAGTTCCTCCTCCC

Figure S1. Electrophoretogram for isolating CDs of *TsGPX3*, *TsGPX5* and *TsGPX7*. (**A**) Totle RNA; (**B**) cDNA quality testing; (**C**) 3' RACE for *TsGPX3*, *TsGPX5*; (**D**) 3' RACE for *TsGPX7*, 71 and 72: PCR product using inner primers; 73 and 74: PCR product using outer primers.

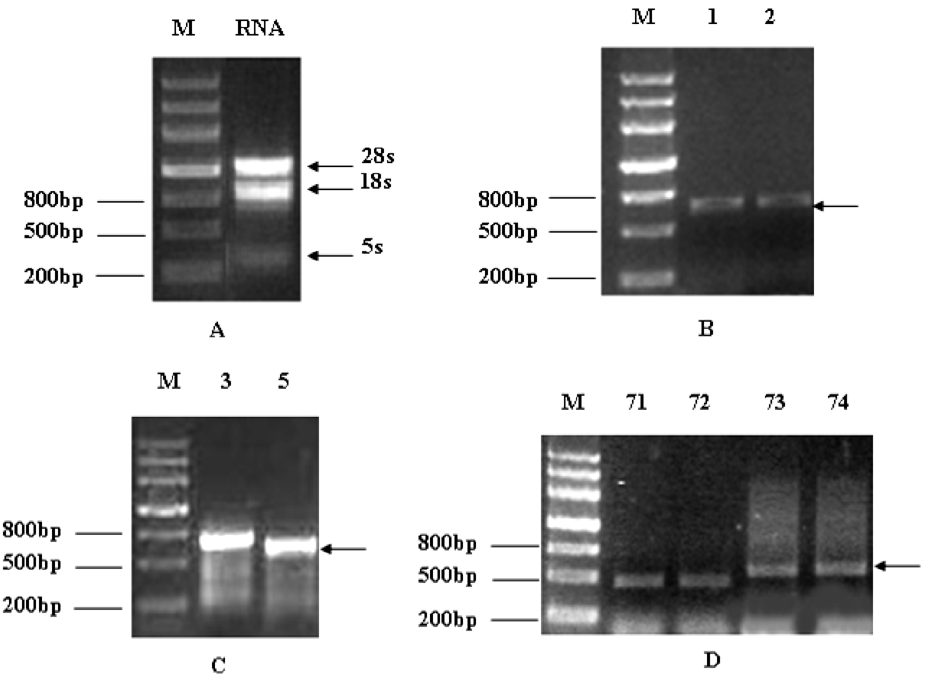

Figure S2. The relative water contents of the *Thellungiella salsuginea* leaves during PEG 6000 treatment. Data are means ± SE of at least six replicates.

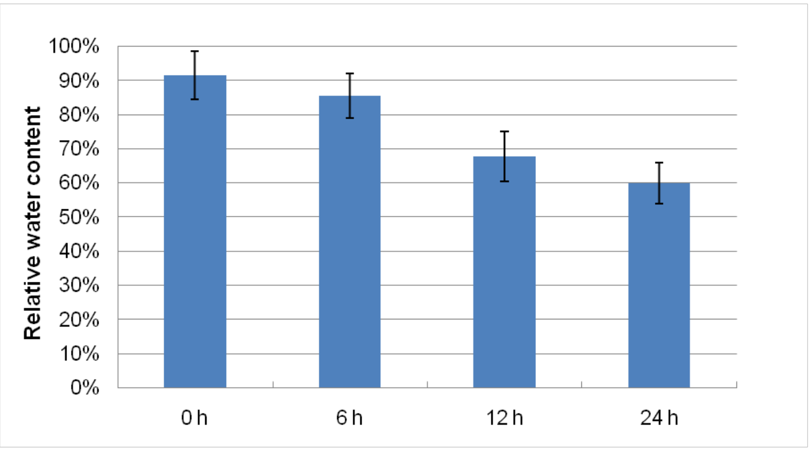

Figure S3. The water potentials of the *Thellungiella salsuginea* leaves during NaCl treatment. Data are means ± SE of at least eight replicates.

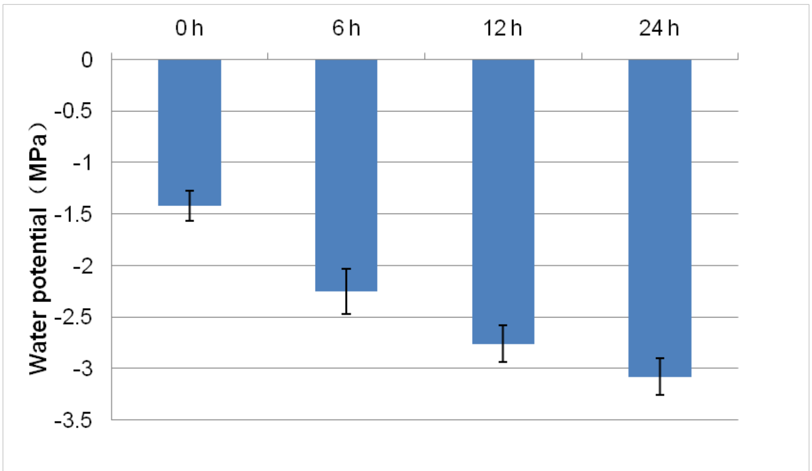

© 2014 by the authors; licensee MDPI, Basel, Switzerland. This article is an open access article distributed under the terms and conditions of the Creative Commons Attribution license (http://creativecommons.org/licenses/by/3.0/).
